# A phase I dose-escalation, safety/tolerability, and preliminary efficacy study of the intratumoral administration of GEN0101 in patients with advanced melanoma

**DOI:** 10.1007/s00262-021-03122-z

**Published:** 2022-01-05

**Authors:** Eiji Kiyohara, Atsushi Tanemura, Kazuma Sakura, Toshihiro Nakajima, Akira Myoui, Naoya Yamazaki, Yoshio Kiyohara, Ichiro Katayama, Manabu Fujimoto, Yasufumi Kaneda

**Affiliations:** 1 Department of Dermatology, Course of Integrated Medicine Graduate School of Medicine, Osaka University, 2-2, Yamadaoka, Suita-shi, Osaka, 565-0871 Japan; 2 Medical Center for Translational Research, Osaka University Hospital, 2-2, Yamadaoka, Suita-shi, Osaka, 565-0871 Japan; 3 GenomIdea Inc, 1-8-31, Midorigaoka, Ikeda shi, Osaka, 563-0026 Japan; 4 Department of Dermatologic Oncology, National Cancer Center Hospital, 5-1-1 Tsukiji, Chuo-ku, Tokyo, 104-0045 Japan; 5 Department of Dermatology, Shizuoka Cancer Center, 1007 Shimonagakubo, Nagaizumi-cho, Sunto-gun, Shizuoka 411-8777 Japan; 6 Vice President Office, Osaka University, 1-1, Yamadaoka, Suita-shi, Osaka, 565-0871 Japan

**Keywords:** Melanoma, Hemagglutinating virus of Japan-envelope, Sendai virus, Innate and adaptive immunotherapy, Clinical trial

## Abstract

Despite recent advance in immunotherapy agents, safe new therapies that enhance the effects of immune checkpoint inhibitors are still required to develop. We previously demonstrated that hemagglutinating virus of Japan-envelope (HVJ-E) induced not only direct tumor cell death but also antitumor immunity through the activation of T and natural killer (NK) cells, thereafter, developed a manufacturing process of HVJ-E (GEN0101) for clinical use. We here performed a phase Ia clinical trial of intratumoral GEN0101 administration in six patients with stage IIIC or IV malignant melanoma. The primary aim was to evaluate the safety and tolerability of GEN0101, and the secondary aim was to examine the objective tumor response. Patients were separated into two groups (*n* = 3 each) and received a low dose of 30,000 and high dose of 60,000 mNAU of GEN0101. All patients completed a two-week follow-up evaluation without severe adverse events. The overall response rate was 33% (2 of 6), with 2 partial responses in the high-dose group and 2 with stable disease, and 2 with progressive disease in the low-dose group. Local complete or partial responses were observed in 11 of 18 (61%) target lesions. One patient demonstrated shrinkage of lung metastases after the treatment. The activity of NK cells and interferon-γ levels were increased in the circulation, indicating augmentation of antitumor immunity by GEN0101. This trial showed not only the safety and tolerability but also the significant antitumor effect of GEN0101, suggesting that GEN0101 might be a promising new drug for patients with advanced melanoma.

**Supplementary Information:**

The online version contains supplementary material available at 10.1007/s00262-021-03122-z.

## Introduction

Advanced melanoma is refractory to anticancer drugs, and many new immunotherapies have been developed in response to this in recent years. Of note, while programmed cell death protein-1 (PD-1) and cytotoxic T lymphocyte antigen-4 (CTLA-4) inhibitors are superior to chemotherapy in terms of their clinical effectiveness for advanced melanoma patients, combination therapy with anti-PD-1 and anti-CTLA4 antibodies can cause severe immune-related adverse events, which are sometimes life-threatening [1]. Therefore, new drugs with few side effects that improve the efficacy of immune-checkpoint inhibitors are urgently needed. As candidates, we focused on the clinical usefulness of intratumoral oncolytic virotherapy instead of systemic therapy.

Hemagglutinating virus of Japan (HVJ), also known as Sendai virus, belongs to the Paramyxovirus family with single-strand RNA [2]. HVJ-envelope (HVJ-E), an inactivated form of HVJ, was developed as a drug delivery vector with membrane fusion activity [3]. The envelope itself has an antitumor effect through the induction of direct killing of cancer cells and activating CD8-positive cells and natural killer (NK) cells [4], 5. In addition, induced tumor-specific cytotoxic T lymphocytes (CTLs) were maintained even when dacarbazine was used in combination with HVJ-E for melanoma [6]. Fragmented RNA in HVJ-E can be taken up into cells by membrane fusion and induce type-1 interferon via the RIG-I pathway [7]. Not only apoptosis but also necroptosis was caused by HJV-E in neuroblastoma [8]. Since HVJ-E exerts an antitumor effect via the above diverse mechanism, we performed a first-in-human phase I/IIa dose-escalation clinical study for patients with advanced melanoma that could be treated intratumorally by HVJ-E at 1000 and 3000 milli neuraminidase units (mNAU) [9]. As a result, the safety and tolerability were confirmed with no dose-limiting toxicity (DLT). CD8^+^ and CD4^+^ cells were recruited to the tumor by HVJ-E. Tumor shrinkage was found at the directly treated and untreated sites. Furthermore, the appearance of vitiligo reflecting the results of the immune response was observed. This result indicated that HVJ-E can activate tumor immunity and may be effective in combination with immune-checkpoints inhibitors. Subsequent to this favorable result, we planned to use higher doses of HVJ-E (GEN0101) at 30,000 and 60,000 mNAU in patients with stage IIIC-IV advanced melanoma.

In the present phase I clinical trial, we performed intratumoral administration of GEN0101 in two dosages to confirm its safety and tolerability as the main purpose and to assess the antitumor effect as the secondary purpose.

## Materials and methods

### Study population

Patients with any type of stage IIIC or IV advanced metastatic melanoma according to the AJCC 7th edition and tumors resistant to standard therapy or patients who refused standard therapy were eligible for this study. Patient eligibility also required evaluable, measurable, and injectable cutaneous, subcutaneous, or lymph node metastatic tumors that were under 25 cm^2^ in area according to Response Evaluation Criteria in Solid Tumors (RECIST; version 1.1) [10]. Patients were between 20 and 91 years old, with an Eastern Cooperative Oncology Group (ECOG) performance status of 0 or 1, a life expectancy over 12 weeks, and adequate bone marrow, liver, and renal function. Melanoma was diagnosed by histology. Patients with known allergies to components of the study drug, multiple brain metastases, severe complications (e.g. active or uncontrolled chronic infection), a history of other malignant tumors within two years, or active autoimmune diseases as well as pregnant or lactating women were excluded. Patient characteristics are detailed in Supplementary Table 1. Patient 3 in the low-dose group had received two cycles of nivolumab at the dose of 2 mg/kg every 3 weeks (ONO-4538–02) before initiation of this trial.

### Study design

The primary aim of this study was to determine the maximum tolerated dose (MTD) and DLT of GEN0101. The secondary and exploratory aims were to evaluate the antitumor effects of GEN0101 and to investigate peripheral immune response induced by GEN0101, respectively.

This clinical study was designed as a non-randomized, open-label phase I study with standard dose escalation and was performed between 2014 and 2016. A clinical control group was not used. With regard to the dose-escalation design of this clinical study, a traditionally used 3 + 3 design was adopted with reference to the phase I study design in clinical studies for cancer, as described in Clinical Trials in Oncology [11]. To ensure safety, the dosage of the low-dose group was set at 30,000 mNAU. After confirming safety in three cases in the low-dose group, the administration of GEN0101 was performed in three cases in the high-dose group. The maximum dosage in the high-dose group was set at 60,000 mNAU, which was one-third the level and the same level as the no observed adverse effect level (NOAEL) in monkey and rat toxicity studies, respectively. Those studies were carried out by intermittent subcutaneous administration using the same dosage (number and frequency) as in this clinical trial. We set the maximum dose of the present study to less than or equal to the NOAEL observed in rat toxicity studies.

In the standard dose-escalation design, an investigation of increased doses in several stages is necessary. However, the number of patients with malignant melanoma in Japan is small. Furthermore, as we focused on cases with advanced-stage cancer, two dosages were established, considering the possibility of completing a clinical study at multiple centers. Written informed consent for this clinical trial was obtained from all individual participants. The study was registered with the UMIN Clinical Trials Registry (no. UMIN000012943).

### Manufacturing and handling of GEN0101 preparations

HVJ-E product purified in GMP grade for clinical use, named GEN0101, was provided by GenomIdea, Inc. (Osaka, Japan). The GEN0101 product contained the following additives: trehalose, sodium chloride, disodium hydrogen phosphate anhydrous, and potassium dihydrogen phosphate. To prepare 10,000 mNAU/mL of GEN0101 solution, lyophilized GEN0101 was dissolved in 1 mL of water for injection. The prepared GEN0101 solution was stored at 4 °C until administration.

### Administration plan

**Fig. 1:**
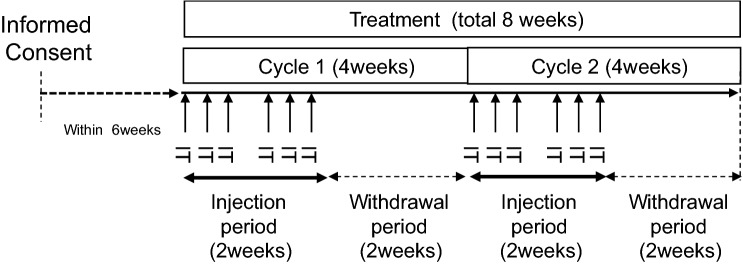
Overview of treatments administered to patients. Black arrows indicate an intralesional administration of GEN0101. Twelve injections were administered over two cycles, and the duration of one cycle was four weeks. The tumor size was evaluated pre-administration and at the end of each cycle

As shown in Fig. 1, GEN0101 was administered three times a week, with at least a one-day interval, for two weeks (i.e. the injection period); this was followed by a two-week withdrawal period. This four-week period in total was regarded as a single cycle. Safety was continuously monitored throughout the study, and the antitumor effects were evaluated at the end of each cycle. The clinical study was completed after two cycles of the treatment (eight weeks).

This clinical study was carried out upon admission of the patients to the hospital. As a general rule, the GEN0101 solution was injected once to three times per target tumor lesion of the skin or lymph node at each session. The number of injections was determined according to the tumor size and depth of target lesion in both groups. The concentration of GEN0101 solution was set as 10,000 mNAU/mL; therefore, 3 mL of 30,000 mNAU and 6 mL of 60,000 mNAU were administered in the low- and high-dose groups, respectively.

### Safety and efficacy assessments

Adverse events were graded according to the National Cancer Institute Common Toxicity Criteria For Adverse Events (NCI-CTCAE, v4.0) [12]. DLT was defined as grade ≥ III nonhematologic toxicity or grade ≥ IV hematologic toxicity. Medical history and demographic data were collected at baseline. A physical examination and monitoring of vital signs were conducted at baseline and throughout the treatment period along with safety assessments (ECOG performance status, 12-leads electrocardiogram). Clinical safety monitoring was carried out throughout the study, and stringent abortion criteria was applied before dose escalation. Tumor responses were assessed at baseline and at every cycle using RECIST version 1.1, with an evaluation of the local tumor size (sum of the maximum length) and occurrence of new lesions. Blood samples were evaluated for immune monitoring, including the NK cell activity and IFN-γ titer, which were analyzed via a Cr-releasing assay and enzyme immunoassay, respectively (LSI Medience Inc., Tokyo, Japan). Statistical significance was not assessed in this study because of the small sample size.

## Results

### Safety and tolerability

Adverse events in which causal relationships could not be ruled out are summarized in Table 1. The total number of adverse events was 224 among the 6 patients, including 3 in the low-dose group and 2 in the high-dose group; in 156 of the 224 adverse events, a causal relationship could not be ruled out. Frequently occurring adverse events in subjective symptoms were injection site reaction, a fever, chills, headache, malaise, and anorexia of grade < II. Frequently occurring adverse events observed as abnormal changes in laboratory test values were a decreased white blood cell count, decreased neutrophil count, and hypocalcemia. The only grade III immune-related adverse event was a decreased neutrophil count in one patient, who recovered within four days. All of these phenomenas were transient. Abnormalities in the other laboratory findings, including vital signs, electrocardiogram, thoracic X-ray, and fluorodeoxyglucose-positron emission tomography results, related to GEN0101 were not observed. As mentioned above, no DLT was observed in either group. Thus, the intratumor injection of high-dose GEN0101 (60,000 mNAU) was generally well-tolerated and safe in patients with advanced melanoma.

**Table 1:** Summary of main adverse events

	AE, All grades n (%)				AE, All grades n (%)		
	Low dose *N* = 3	High dose *N* = 3	Total N = 6		Low dose *N* = 3	High dose *N* = 3	Total *N* = 6
Fever	3 (100)	2 (67)	5 (83)	Decreased white blood cell count	1 (33)	1 (33)	2 (33)
Malaise	2 (67)	2 (67)	4 (67)	Decreased neutrophil count	0 (0)	2 (67)GIII: 1	2 (33)
Injection site pain	1 (33)	2 (67)	3 (50)	Hypo calcemia	0 (0)	2 (67)	2 (33)
Injection site redness/swellin	1 (33)	2 (67)	3 (50)	Increased γGTP	1 (33)	0 (0)	1 (17)
Injection site reaction	2 (67)	0 (0)	2 (33)	Anemia	1 (33)	0 (0)	1 (17)
Injection site anemia	1 (33)	1 (33)	2 (33)	Decreased platelet	0 (0)	1 (33)	1 (17)
Skin ulcer	0 (0)	2 (67)	2 (33)	Hepatic dysfunction	0 (0)	1 (33)	1 (17)
Rigors/chills	1 (33)	1 (33)	2 (33)	Increased hapto globin	0 (0)	1 (33)	1 (17)
Stomatitis	1 (33)	1 (33)	2 (33)				
Dry skin	0 (0)	2 (67)	2 (33)				
Worsened left buttock pain	0 (0)	1 (33)GIII: 1	1 (17)				

*AEs*, adverse events; *GIII*, grade 3 adverse event; *CRP*, C-reactive protein

### Tumor response

All patients received intratumoral injection of GEN0101 in all target lesions. The antitumor effects, evaluated using RECIST version 1.1, including the presence of new lesions, are shown in Table 2. A comprehensive evaluation of the best overall response showed partial response (PR; 33%), stable disease (SD; 33%), and progressive disease (PD; 33%, one patient had PD due to the occurrence of a new lesion), whereas the best evaluation of the sum of target lesion sizes showed PR 50%, SD 33%, and PD 17%.

**Table 2:** Assessment of overall response

		Cycle 1			**Cycle 2**			
	Dose	Target lesions	New lesion	Overall response	Target lesions	New lesion	Overall response	Best overall response
PT 1	Low dose	PR	+	PD	PR	+	PD	PD
PT 2	(30,000 mNAU)	PD	–	PD	PD	–	PD	PD
PT 3		SD	–	SD	SD	–	SD	SD
PT 4	High dose	PR	–	PD	PR	–	PR	PR
PT 5	(60,000 mNAU)	SD	–	SD	PR	–	PR	PR
PT 6		SD	–	SD	SD	–	SD	SD

*SD*, stable disease; *PR*, partial response; *PD*, progressive disease

**Fig. 2:**
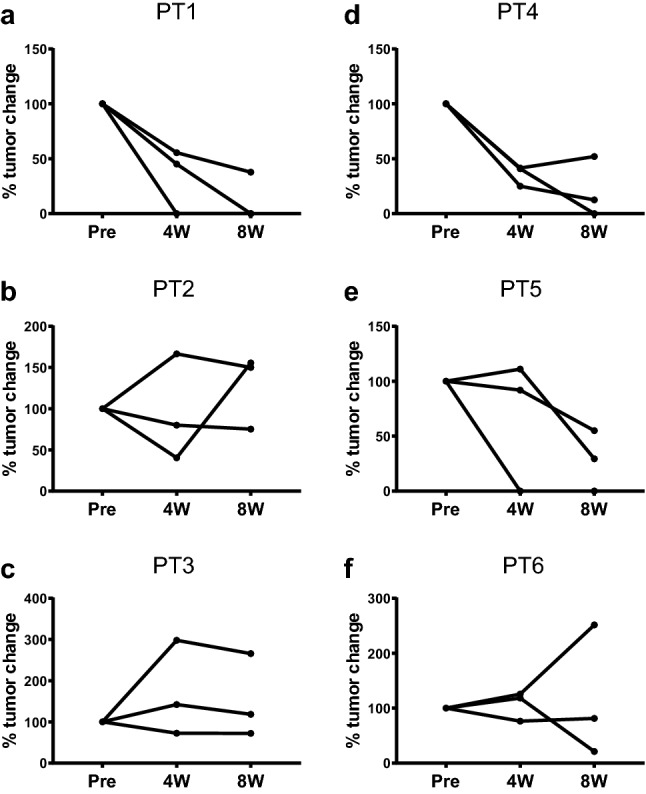
Change in tumor size in six patients. Patient 1 **a**, patient 2 **b**, patient 3 **c**, patient 4 **d**, patient 5 **e** and patient 6 **f**. PT: patient

**Fig. 3:**
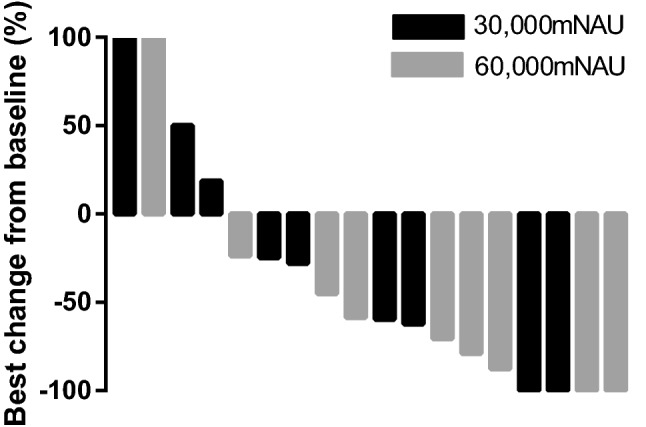
Waterfall plot of the best objective response for target lesions in six patients. Black bars: 30,000 mNAU in the low-dose group. Gray bars: 60,000 mNAU in the high-dose group

**Fig. 4:**
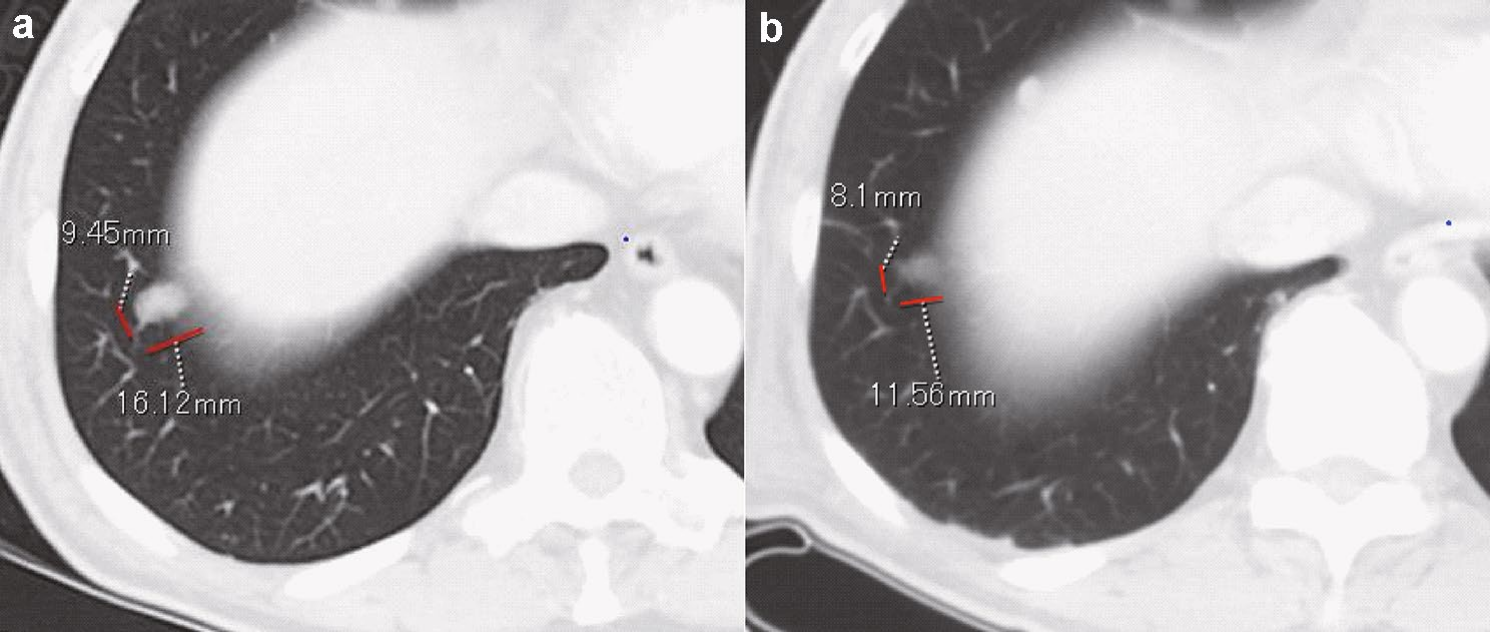
The decreased size of lung metastasis after the administration of GEN0101. The solid lesion in the right pulmonary S9 segment detected at baseline **a** had shrunk at week 8 **b**

In our evaluation of each target lesion, regression of the tumor was observed at the end of study; a complete response (CR) and PR were observed in 4 of 9 lesions (44%) in the low-dose group and 7 of 9 lesions (77.8%) in the high-dose group (Table 3). In total, 11 of 18 (61%) target lesions showed CR and PR in both groups. The response kinetics in the target lesions in each case was observed at 0 (baseline), 4, and 8 weeks (Fig. 2a-f). Thus, local injection with GEN0101 suppressed tumor growth in patients with melanoma in a dose-dependent manner. Waterfall plots of best overall response recorded for all patients in this clinical trial are shown in Fig. 3. In addition, the solitary lesion in the right pulmonary S9 segment of patient 1 in low-dose group, who had failed prior chemotherapy (Fig. 4a), showed 23.1% shrinkage, which was regarded as stable disease according to RECIST version 1.1 (Fig. 4b).

**Table 3:** Tumor response in target lesions

	CR	PR	SD	PD	CR + PR	Total
Low dose, n (%)	2 (22)	2 (22)	3 (33)	2 (22)	4 (44)	9 (100)
High dose, n (%)	2 (22)	5 (56)	1 (11)	1 (11)	7 (78)	9 (100)
Total, n (%)	4 (22)	7 (39)	4 (22)	3 (17)	11 (61)	18 (100)

*CR*, complete response; *PR*, partial response; *SD*, stable disease; *PD*, progressive disease

### Cytokine dynamics during the study

**Fig. 5:**
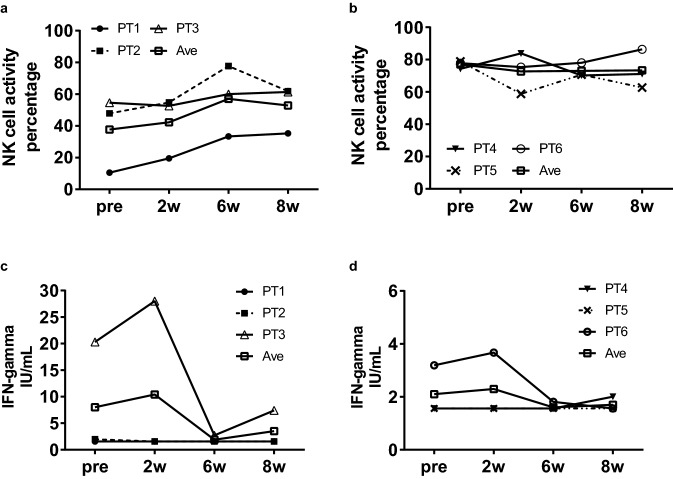
The comparison of the NK cell activity and IFN-γ level of peripheral blood in the six patients between baseline and two, six, and eight weeks. The NK cell activity in patients 1, 2, and 3 **a**; the NK cell activity in patients 4, 5, and 6 **b**; the IFN-γ levels in patients 1, 2, and 3; **c** the IFN-γ levels in patients 4, 5, and 6 **d**

In terms of tumor immunity induction, the NK cell activity and interferon-γ (IFN-γ) levels in the peripheral blood were evaluated at 0, 2, 6, and 8 weeks. GEN0101 showed the trend of increased NK cell activity in patients 1, 2, and 3 (Fig. 5a, b). Finally, the NK cell activity was increased in 67% of patients (patients 1, 2, 3, and 6) at 8 weeks compared with that at 0 weeks. IFN-γ levels were increased in 33% of patients (patients 3 and 6) at 2 weeks (Fig. 5c, d). However, IFN-γ did not increase immediately after the administration of GEN0101 at 6 weeks in the second cycle.

## Discussion

In this clinical study, the safety and tolerability of the intratumoral injection of GEN0101 at two different doses in patients with advanced melanoma were demonstrated. Local regression of the tumor at injection sites and regression of the metastatic tumor in lung was suggested to be mediated by strong antitumor immunity. Thus, the treatment with GEN0101 has been suggested to induce systemic as well as local antitumor immunities, as described below. These data suggest that GEN0101 may represent a new treatment for patients with advanced melanoma.

Notably, there were no serious adverse events in which a causal relationship could be ruled out. DLT was not observed in this trial. Adverse events of CTCAE grade ≥ II, including injection site reactions, occurred due to GEN0101 administration; however, all of these were temporary. The adverse events observed in this study were also already reported in the HVJ-E clinical study and for other intralesional immunotherapies [9] [13]. In addition, no DLT was observed at the same dose of GEN0101 in another clinical trial for prostate cancer [14]. A local skin reaction at the injection site may reflect a substantial immune response to GEN0101. This adverse event was considered to result from the induction of recruitment of immune cells, including CD8^+^ T cells, as was observed in a previous clinical study for melanoma [9]. The decreased neutrophil count observed in this study was suggested to be due to the chemokine-mediated recruitment of neutrophils from bloodstream to tumor tissues. Intratumoral injection of HVJ-E reportedly induces the expression of CXC motif ligand 2, which is a chemokine for neutrophil, and infiltration of N1 neutrophil into tumor tissue in mouse model of melanoma [15]. N1-type neutrophils have an antitumorigenic phenotype in tumor microenvironment [16]. Erythema and heat due to local inflammation repeatedly appeared but receded over time. In addition, a transient fever was able to be suppressed by premedication, such as non-steroidal anti-inflammatory drugs (NSAIDs) and acetaminophen. The fever was considered to have been induced by cytokines, such as interferon-γ. Since a dose of 60,000 mNAU was confirmed to be safe, this dose will likely continue to be used in future clinical trials.

The antitumor effect was comprehensively evaluated based on RECIST as in the clinical study. The results showed an objective response rate (ORR) of 33.3% (2/6) in the total group and 66.7% (2/3) in the high-dose group. This was better than that of previously performed clinical studies with lower doses of GEN0101 (3000 and 10,000 mNAU) [9]. In addition, the tumor response also showed dose-dependency with intratumoral administration; the response rate of the target lesions increased from 44% in the low-dose group to 78% in the high-dose group, suggesting that the intratumoral administration of GEN0101 increased a response rate in a dose-dependent manner.

Intralesional Bacillus Calmette-Guerin (BCG) treatment has been established as immunotherapy for malignant melanoma. The long-term survival of stage IV melanoma patients in the extended MMAIT-IV trial showed a median OS and 5- and 10-year survival of 39.1 months and 43.3 and 33.3%, respectively, with BCG plus placebo [17]. Morton et al. reported that 90% of injected lesions achieved CR, whereas 17% of non-injected lesions showed regression [18]. Furthermore, 30% of patients remained disease free for 6–74 months. BCG demonstrated significant potential as intratumor immunotherapy for malignant melanoma. However, severe complications, such as ulceration, skin necrosis, and abscess, have been reported following repeated injection with BCG [19]. The severe skin complications, in particular, have been a major problem for melanoma patients, hampering their ability to safely receive intralesional BCG therapy. T-VEC, which is also intratumorally administered, like BCG and GEN0101, exerts an antitumor effect by expressing GM-CSF and has been approved by the FDA for malignant melanoma. A phase III clinical trial of T-VEC alone for melanoma patients demonstrated 31.5% of ORR (CR; 16.9%, PR; 14.6%) and 19.3% of durable response rate [20]. An overall survival and a median time to CR were 23.3 (19.5–29.6) and 8.6 months, respectively. The ORRs of lesions injected by T-VEC and GEN0101 in this study were equivalent in 67.2 and 61%, respectively [21]. The effect of high-dose GEN0101 may be comparable to that of T-VEC, as the local site response rate in the high-dose group was 78%. Another oncolytic virus therapy, coxsackie A21, was intratumorally administered to patients in a phase II trial of 57 stage IIIC-IVM1c melanoma patients [22]. A total of 38.6% of patients achieved an immune-related progression free survival of 6 months. The ORR was 28.1% in that trial. High-dose GEN0101 appears to exert a similar antitumor efficacy to these oncolytic viruses.

In addition to direct killing, antitumor immunity induced by GEN0101 may be exerted as therapeutic effect. HVJ-E recruits CD8^+^ T cells at the administration site as well as at the non-administration site to evoke antitumor immunity [9]. Therefore, it is considered that antitumor immunity was also induced by GEN0101 in the present trial; an increase in the NK cell activity and IFN-γ was confirmed (Fig. 5). The NK cell activity increased immediately after the administration of GEN0101, peaking at 6 weeks and followed by a gradual decrease in the low-dose group. This tendency was also seen during clinical studies, suggesting that the NK cell response was transient. There was little change in the NK cell activity in the high-dose group, possibly because of the high level of NK cell activity (about 80%) at baseline. IFN-γ levels increased transiently after six administration sessions at two weeks, which was considered to contribute to the activation of CTL. According to an in vitro study, HVJ-E can induce dendritic cell maturation [4]. Tumor-specific T cells were trained by dendritic cells infiltrated from local tumor [4]. CTL against local tumor antigen was also detected in a mouse malignant melanoma model [6]. Based on these findings, we suspect that activated NK cells and CTLs stimulated by the production of IFN-γ spread to distant metastasis and might have thus contributed to the reduced size of lung metastasis (Figs. 4 and 5). Systemic effects of HVJ-E may be mediated by both innate and adaptive immunity in humans. Therefore, the more potent efficacy may be obtained via combination therapy with an immune checkpoint inhibitor.

In terms of immune induction, T-VEC induces CD8^+^ CTL activity [23]. In addition, CD8^+^ T cells existing around the tumor have been reported to be involved in a favorable response to the treatment with anti-PD-1 antibodies in patients with malignant melanoma [24]. Therefore, combination therapies of T-VEC and immune checkpoint inhibitors have been attempted in order to improve the therapeutic effect. In phase II clinical trial of T-VEC in combination with ipilimumab, an anti-CTLA4 antibody, the response rate was 39% in the combination group and 18% in the ipilimumab-alone group, with a significant difference. In that clinical trial, ipilimumab was injected after the intratumoral administration of T-VEC [25]. Intralesional BCG followed by ipilimumab was not tolerated in advanced melanoma and showed no evidence of clinical benefit [26]. In another clinical trial, the combination of T-VEC and ipilimumab showed an ORR of 50% in patients with melanoma [27]. A phase Ib clinical trial was also conducted in combination with another immune checkpoint inhibitor (the anti-PD-1 antibody Pembrolizumab), targeting untreated patients with stage IIIB to IV melanoma [28]. In that clinical trial, an ORR was 61.9%, with 33.3% showing CR. An over 50% reduction in tumor volume was observed in 82% of injected lesions. Interestingly, the response was correlated not with the baseline number of CD8^+^ cells around the tumor but instead with the intratumoral infiltration of CD8^+^ cells after the administration of T-VEC. In Phase II, single-arm, biomarker study of T-VEC monotherapy, T-VEC increased the ratio of CD3^+^/CD8^+^ T cells expressing granzyme B, CTLA-4, and PD-1 [29]. Such mechanism may be one reason for the high response rate obtained in combination therapy. Similar to T-VEC, GEN0101 was predicted to induce the dense intratumoral infiltration of CD4^+^ and CD8^+^ T cells, which may have contributed to tumor cell death, according to previous clinical study [9]. Therefore, GEN0101 combined with immune checkpoint inhibitors may enhance the clinical effectiveness against malignant melanoma. In particular, since GEN0101 was originally developed as a vector, entities such as cytokine genes, anticancer agents, and siRNA are expected to be able to be enclosed in GEN0101, which will induce the more efficient antitumor effect.

In summary, the results of this clinical trial suggest that GEN0101 at 30,000 and 60,000 mNAU is tolerable and effective for the treatment of melanoma patients. The response rates of target lesions in the low- and high-dose groups were 44 and 78%, respectively. This result implies that the antitumor effects on cutaneous target lesions increase in a dose-dependent manner. Furthermore, antitumor immunity in lung metastatic lesion was induced by the local injection of GEN0101. To investigate the antitumor immunity induced by GEN0101 in combination with the immune checkpoint inhibitors, we started a phase Ib/II clinical trial of the combination of GEN0101 and pembrolizumab in patients with advanced melanoma. We expect this phase Ib/II clinical trial to show an improvement in the antitumor effect of GEN0101.

### Supplementary Information

Below is the link to the electronic supplementary material.

Supplementary file1 (PDF 253 kb)

## Data Availability

Not applicable.

## Abbreviations

BCG: Bacillus Calmette-Guerin
CR: Complete response
CTLA4: Cytotoxic T lymphocyte antigen 4
DLT: Dose-limiting toxicity
ECOG: Eastern Cooperative Oncology Group
HVJ: Hemagglutinating virus of Japan
HVJ-E: HVJ-envelope
mNAU: Milli neuraminidase unit
MTD: Maximum tolerated dose
NCI-CTCAE: National Cancer Institute Common Toxicity Criteria For Adverse Events
NOAEL: No observed adverse effect level
ORR: Objective response rate
PD: Progressive disease
PD-1: Programmed cell death protein 1
PR: Partial response
RIG-I: Retinoic acid-inducible gene-I
SD: Stable disease
