# Supplementary material for: A phase I dose-escalation, safety/tolerability, and preliminary efficacy study of the intratumoral administration of GEN0101 in patients with advanced melanoma
Source: Cancer Immunol Immunother. 2022 Jan 5;71(8):2041–9. doi: 10.1007/s00262-021-03122-z (PMC9293878; doi:10.1007/s00262-021-03122-z)
Supplement: Supplementary file 1 — Supplementary file1 (PDF 253 kb) [file 262_2021_3122_MOESM1_ESM.pdf]

Supplementary table 1 Patient characteristics

Characteristic		No.	Ratio (%)
Patients enrolled		6	
Age years	Average	67(63-79)	
Sex	male	4	66.7
	female	2	33.3
ECOG	0	5	83.3
	1	1	16.7
Stage	IIIc	2	33.3
	IV M0	0	0
	IV M1a	1	16.7
	IV M1b	0	0
	IV M1c	3	50
Status of Metastasis	lymph node	4	66.7
	lung	2	33.3
	bone	1	16.7
	skin&subcutaneous tissue	3	50
	other tissue	3	50
Baseline LDH	<ULN	6	100
(Upper limit of normal: ULN)	>ULN	0	0
Type of previous therapy	chemotherapy	3	50
	immunotherapy*, local interferon	4	66.7
	surgery	6	100
	radiation	2	33.3

* One patient was received ONO-4538 (Nivolumab) in clinical study
